# Alantolactone suppresses inflammation, apoptosis and oxidative stress in cigarette smoke-induced human bronchial epithelial cells through activation of Nrf2/HO-1 and inhibition of the NF-κB pathways

**DOI:** 10.1186/s12931-020-01358-4

**Published:** 2020-04-22

**Authors:** Xiaomin Dang, Beibei He, Qian Ning, Ya Liu, Jianxin Guo, Gang Niu, Mingwei Chen

**Affiliations:** 1 Department of Respiratory and Critical Care Medicine, The First Affiliated Hospital of Xi’an Jiaotong University, No. 277 Yanta west road, Xi’an, 710061 China; 2 Department of Medical Imaging, The First Affiliated Hospital of Xi’an Jiaotong University, Xi’an, 710061 China

**Keywords:** Chronic obstructive pulmonary disease, Alantolactone, Cigarette smoke extract, Inflammation, Apoptosis, Oxidative stress

## Abstract

**Background:**

It is well established that airway remodeling and inflammation are characteristics for chronic obstructive pulmonary disease (COPD). Moreover, cigarette smoke extract (CSE) promots inflammation, apoptosis and oxidative stress in COPD. And, there is evidence suggested that alantolactone (ALT), a sesquiterpene lactone isolated from *Inula helenium*, plays an adverse role in inflammation, apoptosis and oxidative stress. However, few studies have investigated the function and mechanism of ALT treatment on the COPD pathological process.

**Methods:**

The levels of IL-1 β, TNF-α, IL-6 and IFN-γ were examined by ELISA. Cells’ apoptosis and caspase-3 activity were detected by Cell Death Detection PLUS enzyme-linked immunosorbent assay and caspase-Glo 3/7 Assay, respectively. The content of malondialdehyde (MDA) and superoxide dismutase (SOD) were determined by using MDA and SOD assay kits. Reactive oxygen species (ROS) generation was measured by DCFH-DA assay. Protein expression was assayed by Western blot.

**Results:**

In the present study, we aimed to observe the protective effects of ALT against inflammation, apoptosis and oxidative stress in human bronchial epithelial Beas-2B and NHBE cells. Our results showed that different doses of CSE exposure induced Beas-2B and NHBE cell inflammatory cytokines IL-1 β, TNF-α, IL-6 and IFN-γ expression, cell apoptosis, caspase-3 activity and mediated oxidative stress markers MDA, ROS and SOD levels, while ALT treatment counteracted the effects of CSE. Further studies suggested that ALT attenuated NF-κB pathway activation. ALT also activated the Nrf2/HO-1 signal pathway through promoting Nrf2 nuclear aggregation and downstream HO-1 protein expression. HO-1 inhibitor tin protoporphyrin IX (SnPP IX) reversed the effects of ALT on Beas-2B and NHBE cell inflammation, apoptosis and oxidative stress.

**Conclusions:**

The above results collectively suggested that ALT suppressed CSE-induced inflammation, apoptosis and oxidative stress by modulating the NF-ĸB and Nrf2/ HO-1 axis.

## Background

Chronic obstructive pulmonary disease (COPD) is a serious lung disease and the mortality and morbidity is increasing annually worldwide according to the World Health Organization (WHO) [1]. Evidence has indicated that COPD is characterized by airway progressive obstruction and chronic inflammation, which leads to emphysema and chronic bronchiolitis [2]. The incidence rate of COPD was 6–8% in population with extra-pulmonary disease, such as abnormal autonomic control of cardiopulmonary function, respiratory muscle weakness as well as cardiac and cardiovascular autonomic regulation diseases [3–5]. Accumulating evidence suggests that cigarette smoking (CS) can affect the immune system and act as a main factor of COPD [6, 7]. Despite the fact that some therapeutic methods, such as phosphodiesterase-4 inhibitors and surgical therapy are used to limit COPD exacerbation, reduce airway obstruction and improve the quality of COPD patient’s life, the therapeutic effect is not satisfactory [8, 9].

*Inula helenium* possessed anti-inflammatory activity in cultured human respiratory epithelium and human neutrophils [10]. The natural sesquiterpene lactone alantolactone (ALT) was isolated from *Inula helenium L.* and *Inula japonica*, and there has been ample evidence to suggest that the compound possesses a wide range of biological activities, including anti-allergic, anti-bacterial, anti-microbial, anti-fungal, anti-oncogenic, anti-helminthic, hepatoprotective and neuroprotective activities [11, 12]. Also, Wang and his colleagues confirmed that ALT contributed to inflammation, oxidative stress and apoptosis pathways in rats with traumatic brain injury [12]. Moreover, inflammation, oxidative stress and human bronchial epithelial cell apoptosis were involved in COPD pathogenesis [13, 14]. Thus, we hypothesized that ALT may play roles in COPD and to the best of our knowledge, this compound has not been used for COPD treatment.

Nuclear factor erythroid-2-related factor-2 (Nrf2) is a master transcription factor and belongs to the cap ‘n’ collar subfamily [15]. According to statistics, Nrf2 could mediate genes that encodedrug transporters, anti-oxidative enzymes, anti- apoptotic proteins and detoxifying factors [16, 17]. Moreover, Nrf2 plays a protective role against oxidative stress through regulating anti-oxidative genes, such as NQO1 and heme oxygenase-1 (HO-1) [18]. Moreover, Jiang et al. showed that Nrf2 regulates HO-1 protein transcription in glutamate-induced HT-22 cell ferroptosis [19]. Cheng et al. indicated that cigarette smoke particle-phase extract mediated HO-1 and Nrf2 expression in human tracheal smooth muscle cells [20]. In the present study, we observed that ALT treatment obviously abrogated the inhibition of the Nrf2/HO-1 signal pathway and the activation of NF-κB signal pathway which were induced by cigarette smoke extract (CSE).

In summary, our study confirmed that CSE induced Beas-2B and NHBE cell inflammation, apoptosis and oxidative stress through activation of Nrf2/HO-1 and inhibition of the NF-κB pathways.

## Materials and methods

### Chemicals

**Fig. 1:**
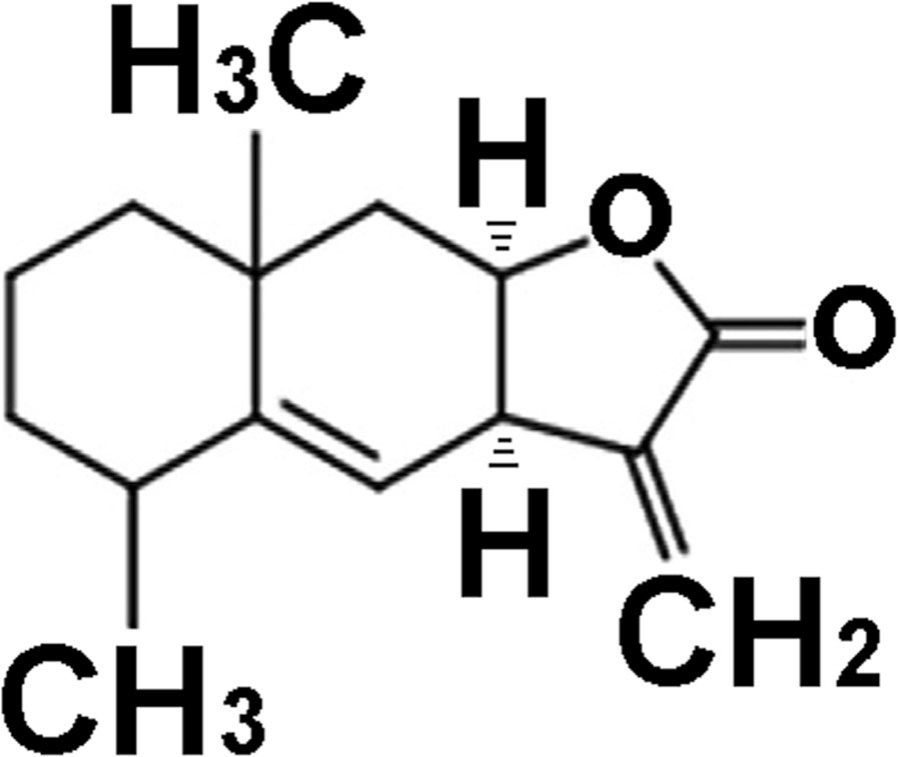
The chemical structure of alantolactone

Alantolactone (ALT, SML0415, molecular weight 232.32) was obtained from Sigma-Aldrich (St. Louis, MO, USA) and the purity (≥98%) which confirmed by HPLC. The chemical structure is shown in Fig. 1. Tin protoporphyrin IX (SnPP IX) was purchased from Porphyrin Products (Logan, UT, USA).

### Cell culture and treatment

Human bronchial epithelial cells Beas-2B (Cat#: CRL-9609) and normal bronchial epithelial cells NHBE (Cat#: CRL-2078) were both purchased from the American Type Culture Collection (ATCC, Manassas, VA, USA). Cells were maintained into RPMI1640 medium (Gibco, New York, NY, USA) added with 10% FBS (Gibco), 100 U/mL penicillin and 100 U/mL streptomycin in a humidified atmosphere under 5% CO_2_ condition at 37 °C. For CSE treatment, human bronchial epithelial cell lines Beas-2B and NHBE cells were maintained in medium without FBS with cigarette smoke extract (CSE) 1, 2 and 5% at the indicated time (24 h). Beas-2B and NHBE cells were pretreated for 2 h with 1, 5 and 10 μM ALT or SnPP (20 μM) for 2 h and then treated with 5% CSE for 24 h.

### CSE preparation

CSE preparation was according to the method described previously with a minor modification [21, 22]. A total of 400 mL of cigarette smoke from commercial Da Qianmen cigarettes (containing 2.5 mg of nicotine and 12 mg of tar per cigarette, Shanghai, China) was drawn into a modified 50 mL syringe apparatus. Each cigarette was completely smoked within 6–8 min. The smoke was mixed with 20 mL serum-free RPMI 1640 medium by vigorous shaking, and this solution, regarded as 100% strength CSE. The solution was adjusted to a pH of 7.4 and then filtered using a 0.22 휇m filter. 100% CSE (100 μl) was used when the value of OD320 nm - OD540 nm between 0.9 and 1.2. CSE solution was diluted with RPMI 1640 medium to indicated concentration and used in experiments within 15 min after preparation.

### Inflammatory cytokines ELISA assay

The levels of IL-1 β, TNF-α, IL-6 and IFN-γ in culture supernatants were examined by the commercially available ELISA kits (R&D Systems, Minneapolis, MN, USA) according to the manufacturers’ instructions.

### Cell death detection method

Beas-2B and NHBE cells were pretreated for 2 h with 1, 5 and 10 μM ALT and then treated with 5% CSE for 24 h. Cell Death Detection PLUS enzyme-linked immunosorbent assay (ELISA; Roche, Nutley, NJ) was used to measure cell apoptosis following the manufacturer’s protocol. The relative cell apoptosis was normalized to the control group.

### Lactate dehydrogenase (LDH) assay

Beas-2B and NHBE cells were pretreated for 2 h with 1, 5 and 10 μM ALT and then exposed with 5% CSE for 24 h. The cell supernatants (100 μL) of each group were collected, and LDH activity was measured by using a commercial LDH Kit (Keygen, Nanjing, China) according to the manufacturer’s instructions. The absorbance at 450 nm wavelength was measured using a microplate reader (Themo Multiskan MK3, USA).

### Caspase-3 activity

Beas-2B and NHBE cells were plated into 96-well plates and pretreated for 2 h with 1, 5 and 10 μM ALT and then treated with 5% CSE for 24 h. Caspase-3 activity was measured by using caspase-Glo 3/7 Assay (Promega, Madison, WI, USA) kit in accordance with a previous report and the manufacturer’s instructions [23]. Capase-Glo 3/7 Reagent (100 μL) was added to cells for another 2 h at room temperature and the luminescence intensity was recorded at 570 nm by using a microplate reader.

### MDA-, SOD- and ROS-level measurement

The content of malondialdehyde (MDA) and superoxide dismutase (SOD) were determined by using MDA and SOD assay kits (Nanjing Jiancheng Bioengineering institute, Jiangsu, China) according to the manufacturer’s instruction. Intracellular reactive oxygen species (ROS) generation was measured by fluorescence dye 2′7’-dichlorofluorescin diacetate (DCFH-DA; Beyotime Institute of Biotechnology, China). Beas-2B and NHBE cells were plated into 12-well plates and pretreated for 2 h with 1, 5 and 10 μM ALT and then exposed with 5% CSE for 24 h. DCFH-DA solution (20 μM) was added to cells for 30 min and the fluorescence was measured by a fluorescence microscope (Invitrogen, WA, USA).

### Western blot

Proteins from Beas-2B and NHBE cells were extracted using the RIPA lysis buffer (Beyotime). Equal amounts of protein (40 μg/lane) were loaded on 10% SDS-PAGE and then transferred to nitrocellulose filter membrane. The membrane was incubated with the following primary antibodies and then incubated with secondary antibodies. The primary antibodies used in this study were: HO-1 (ab13243, Abcam, Dilution: 1:2000); Nrf2 (ab137550, Abcam, Dilution: 1:1000); p-P65 (ab86299, Abcam, Dilution: 1:2000); P65 (ab16502, Abcam, Concentration: 1:5000); and β-actin (ab8227, Abcam, Dilution: 1:2000). The following secondary antibody was used in this study: goat anti-rabbit IgG H&L (HRP, ab205718, Abcam, Dilution: 1:5000). Chemidoc XRS (Bio-Rad, Hercules, CA, USA) was used to detect protein bands.

### Statistical analysis

Data were analyzed using GraphPad prism 5 (GraphPad Software, Inc., La Jolla, CA, USA) and expressed as mean ± standard deviation (SD) from three independent experiments. All statistical analyses were performed through ANOVA test and Bonferonni’s post hoc test. *P* values less than 0.05 were considered to be significantly different.

## Results

### ALT treatment suppressed CSE exposure induced inflammation of Beas-2B and NHBE cells

**Fig. 2:**
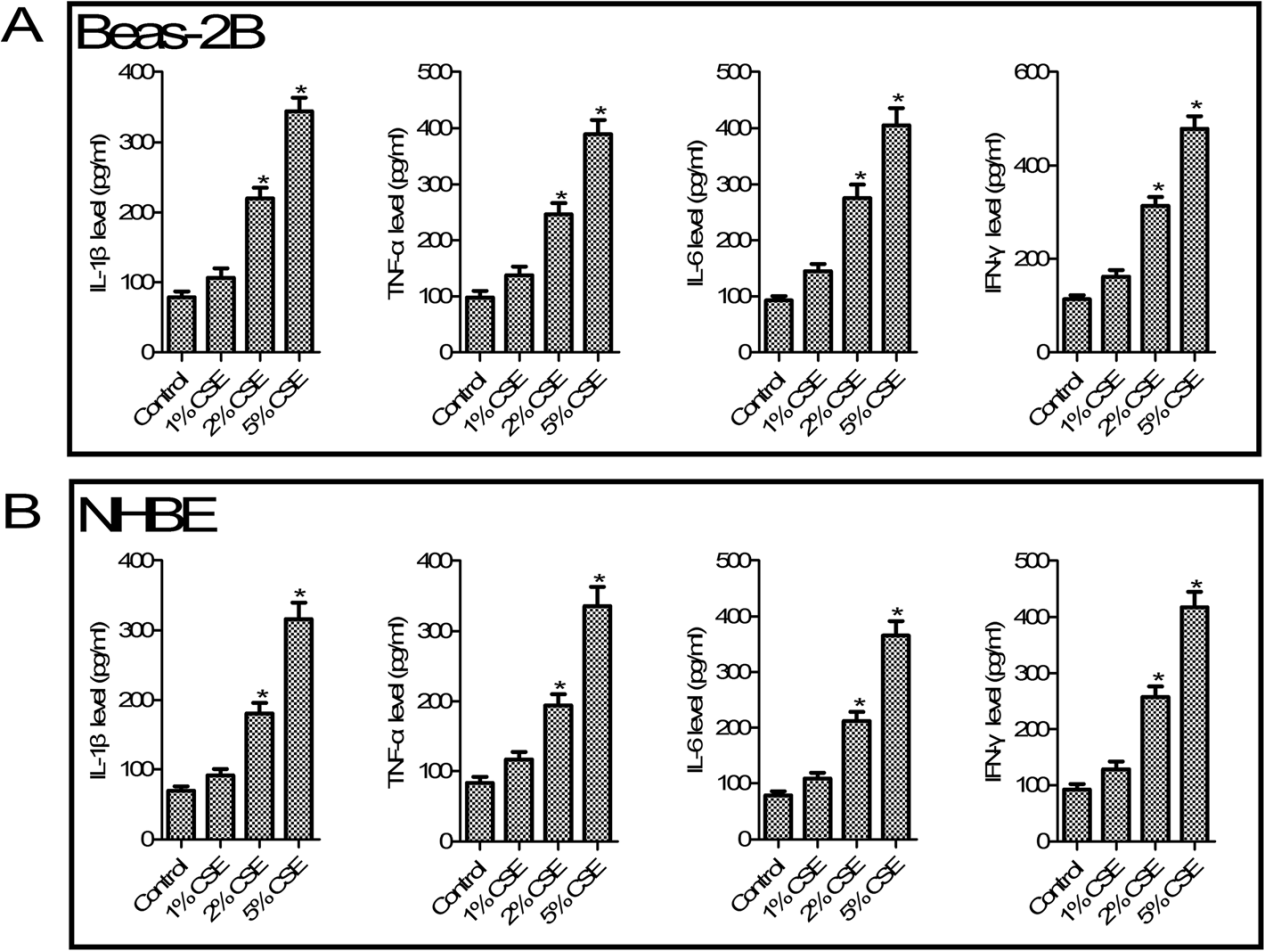
CSE promoted IL-1 β, TNF-α, IL-6 and IFN-γ production in Beas-2B and NHBE cells. **a** CSE increased Beas-2B cell inflammatory response; **b** CSE increased NHBE cell inflammatory response. Beas-2B and NHBE cells were treatment with 1, 2 and 5% CSE for 24 h. Data were expressed as mean ± S.D. from three independent experiments. ^*^P values < 0.05 compared with the control group. CSE: cigarette smoke extract

**Fig. 3:**
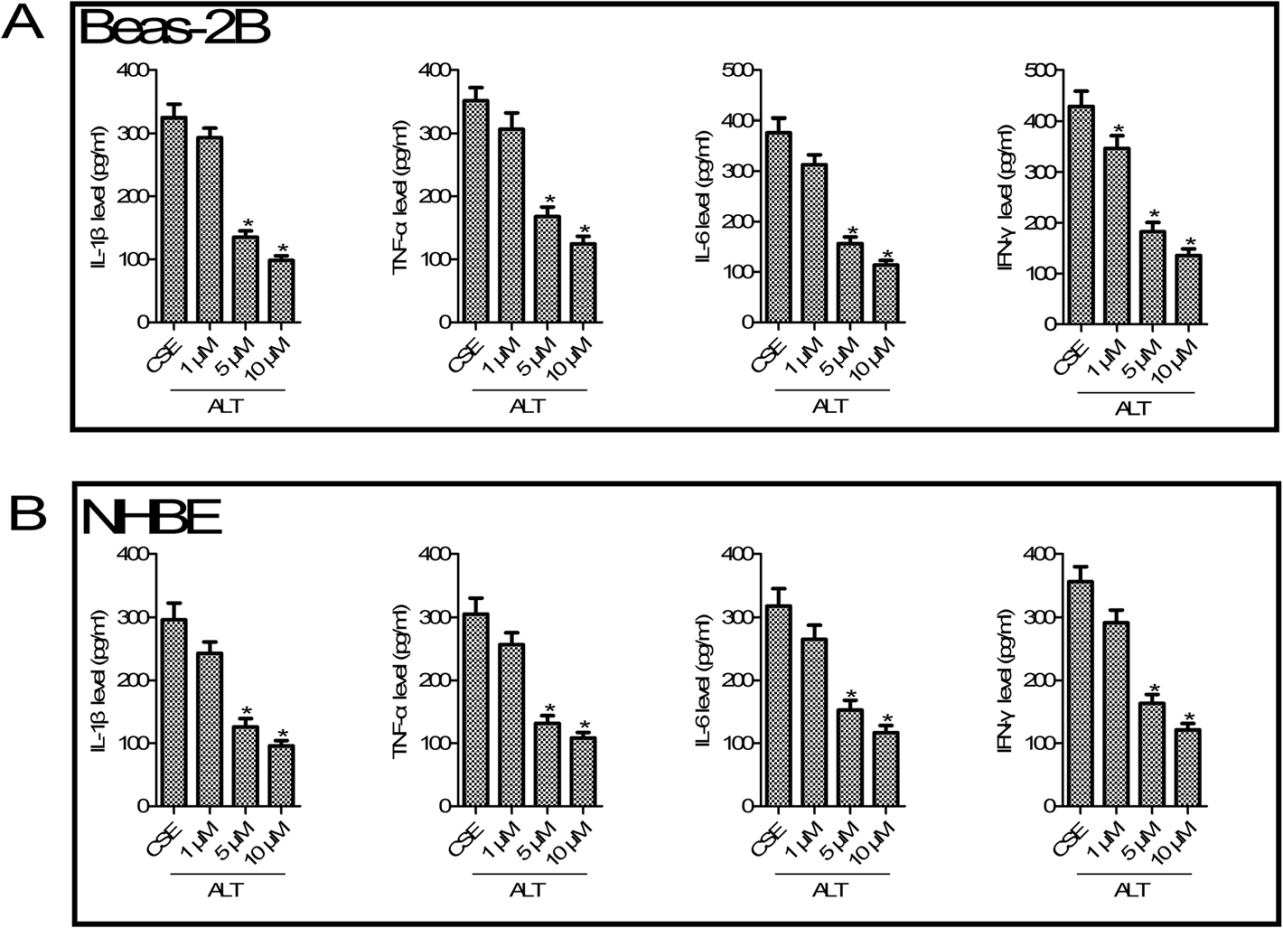
ALT inhibited CSE induced - IL-1 β, TNF-α, IL-6 and IFN-γ production in Beas-2B and NHBE cells. **a** ALT inhibited CSE induced inflammatory response in Beas-2B cells; **b** ALT inhibited CSE induced inflammatory response in NHBE cells. Beas-2B and NHBE cells were pre-treated with 1, 5 and 10 μM ALT for 2 h and then administrated with 5% CSE for 24 h. The commercially available ELISA kits were used to measure the level of IL-1 β, TNF-α, IL-6 and IFN-γ. Data were expressed as mean ± S.D. from three independent experiments. ^*^P values < 0.05 compared with the control group, ^#^*P* values < 0.05 compared with the CSE group. CSE: cigarette smoke extract; ALT: Alantolactone

According to statistics, smoking is a major risk factor of COPD [24]. To mimic in vitro COPD pathological condition, CSE was added to Beas-2B and NHBE cells. As shown in Fig. 2, compared with the control group, different concentrations (1, 2 and 5%) of CSE increased inflammatory cytokines IL-1 β, TNF-α, IL-6 and IFN-γ production at 24 h (all *p* < 0.05) both in Beas-2B and NHBE cells. For further studies, we selected the concentration of CSE at 5%. We next tested the role of ALT in CSE-induced inflammation. As shown in Fig. 3, compared with the CSE exposure group, ELISA analysis of Beas-2B and NHBE cells confirmed a dose-dependent decrease of IL-1 β, TNF-α, IL-6 and IFN-γ secretion after 1, 5 and 10 μM ALT exposure (all *p* < 0.05).

### ALT treatment suppressed CSE exposure induced LDH activity and apoptosis of Beas-2B and NHBE cells

**Fig. 4:**
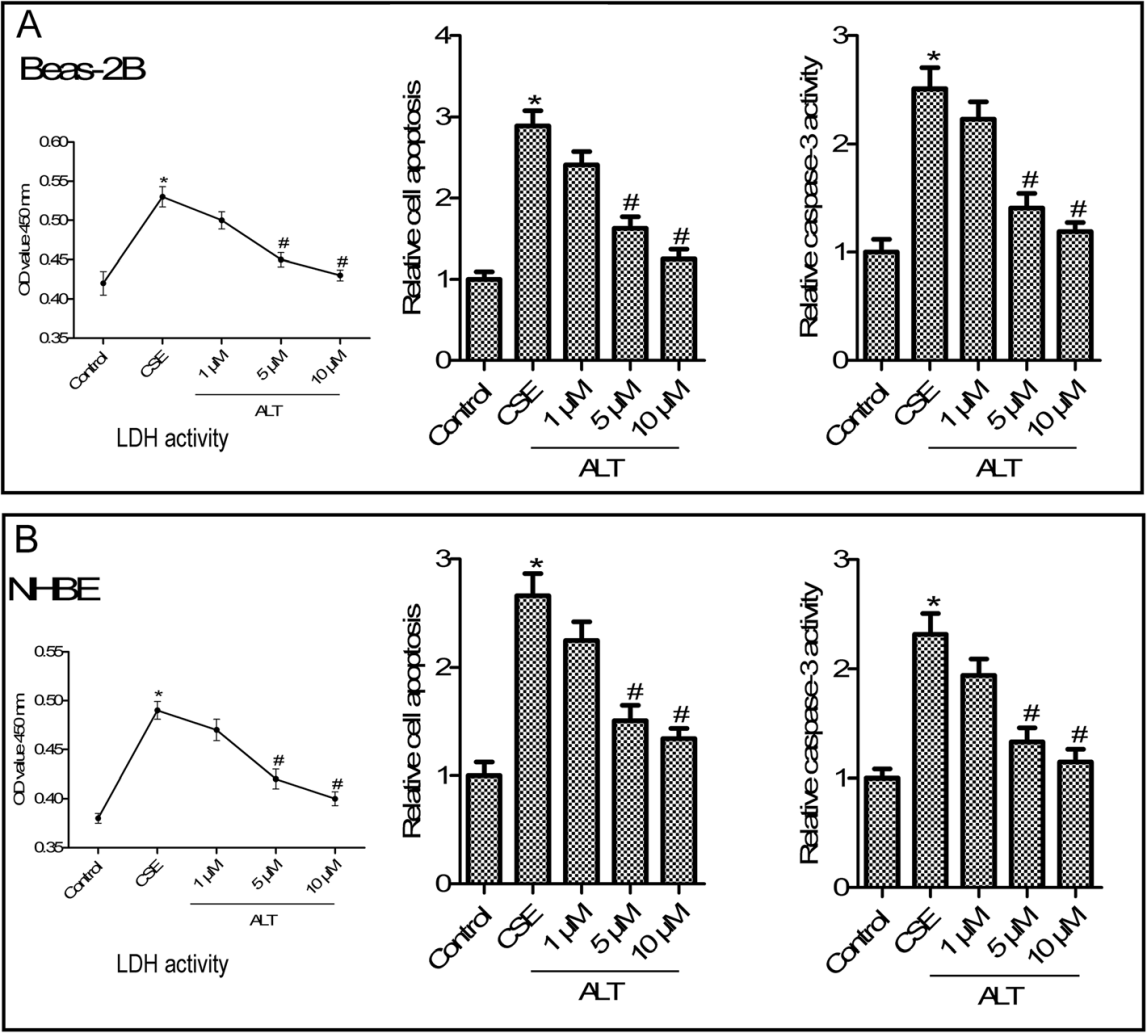
ALT inhibited CSE induced-LDH activity and apoptosis in Beas-2B **a** and NHBE **b** cells. LDH activity was measured using an LDH-cytotoxicity Kit; Cell Death Detection PLUS ELISA assay was used to detect cell apoptosis; Caspase-3 activity was measured by using Caspase-Glo 3/7 assay. Beas-2B and NHBE cells were pre-treated with 1, 5 and 10 μM ALT for 2 h and then administrated with 5% CSE for 24 h. Data were expressed as mean ± S.D. from three independent experiments. ^*^P values < 0.05 compared with the control group, ^#^P values < 0.05 compared with the CSE group. CSE: cigarette smoke extract; ALT: Alantolactone

LDH activity of the supernatant of Beas-2B and NHBE cells was measured using an LDH-cytotoxicity Kit. As shown in Fig. 4 a and b, CSE exposure significantly increased LDH activity in Beas-2B and NHBE cells. The LDH activity was incereased in CSE induced-Beas-2B and NHBE cells, and ALT (1, 5 and 10 μM) treatment reduced LDH activity compared with the CSE group (*p* < 0.05). (Fig. 4 a and b, *p* < 0.05). Because of evidence showing that airway epithelial cell apoptosis was implicated in the pathogenesis of COPD [25], we next examined the role of ALT in CSE-induced Beas-2B and NHBE cells apoptosis. As shown in Fig. 4 a and b, results from a Cell Death Detection PLUS ELISA assay showed that CSE exposure increased Beas-2B and NHBE cells apoptosis, while ALT (1, 5 and 10 μM) treatment distinctly reduced cell apoptosis compared with the CSE group (*p* < 0.05). Caspase-Glo 3/7 assay analysis of Beas-2B and NHBE cells confirmed a marked increase of caspase-3 activity in the CSE group compared with the control group, and a significant reduction of caspase-3 activity in the 1, 5 and 10 μM ALT group compared with the the CSE group (Fig. 4 b, *p* < 0.05).

### ALT inhibited CSE induced oxidative stress in Beas-2B and NHBE cells

**Fig. 5:**
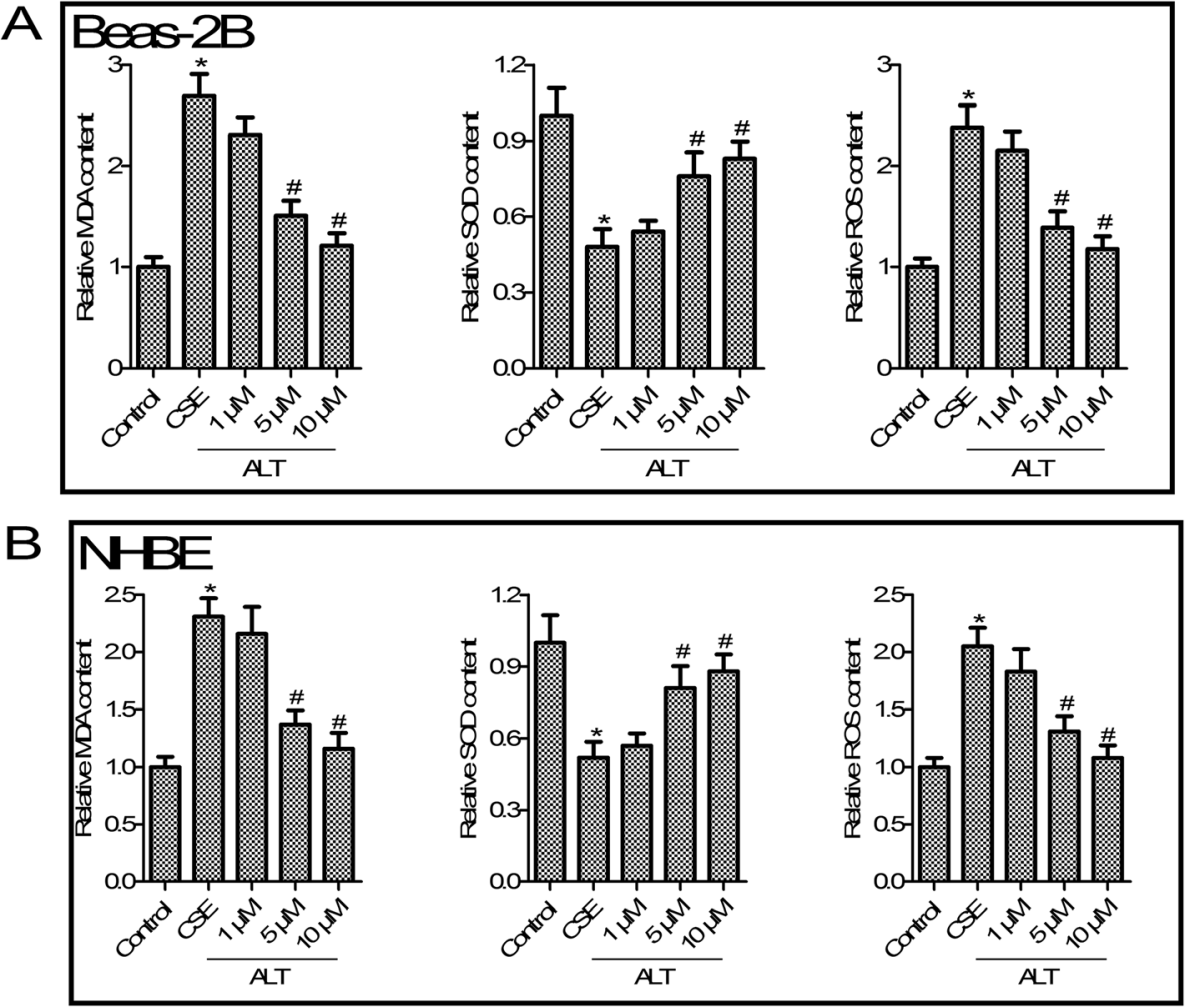
ALT inhibited CSE induced -oxidative stress in Beas-2B **a** and NHBE **b** cells. MDA assay kits was used to measure MDA content; SOD assay kits was used to measure SOD content; DCFH-DA method was used to measure ROS production. Beas-2B and NHBE cells were pre-treated with 1, 5 and 10 μM ALT for 2 h and then administrated with 5% CSE for 24 h. Data were expressed as mean ± S.D. from three independent experiments. ^*^P values < 0.05 compared with the control group, ^#^*P* values < 0.05 compared with the CSE group. CSE: cigarette smoke extract; ALT: Alantolactone

We next determined the effect of ALT on Beas-2B and NHBE cell oxidative stress, compared with the control group, as shown in Fig. 5 a and b, CSE treatment significantly increased MDA content and ROS production, whereas it suppressed the SOD level in Beas-2B and NHBE cells (*p* < 0.05). We next observed that 1, 5 and 10 μM ALT reduced oxidative stress markers’ MDA content and ROS production, and increased the SOD level in Beas-2B and NHBE cells when compared with the CSE group (*p* < 0.05, Fig. 5 a and b).

### ALT treatment suppressed NF-κB pathway activation in CSE induced Beas-2B and NHBE cells

**Fig. 6:**
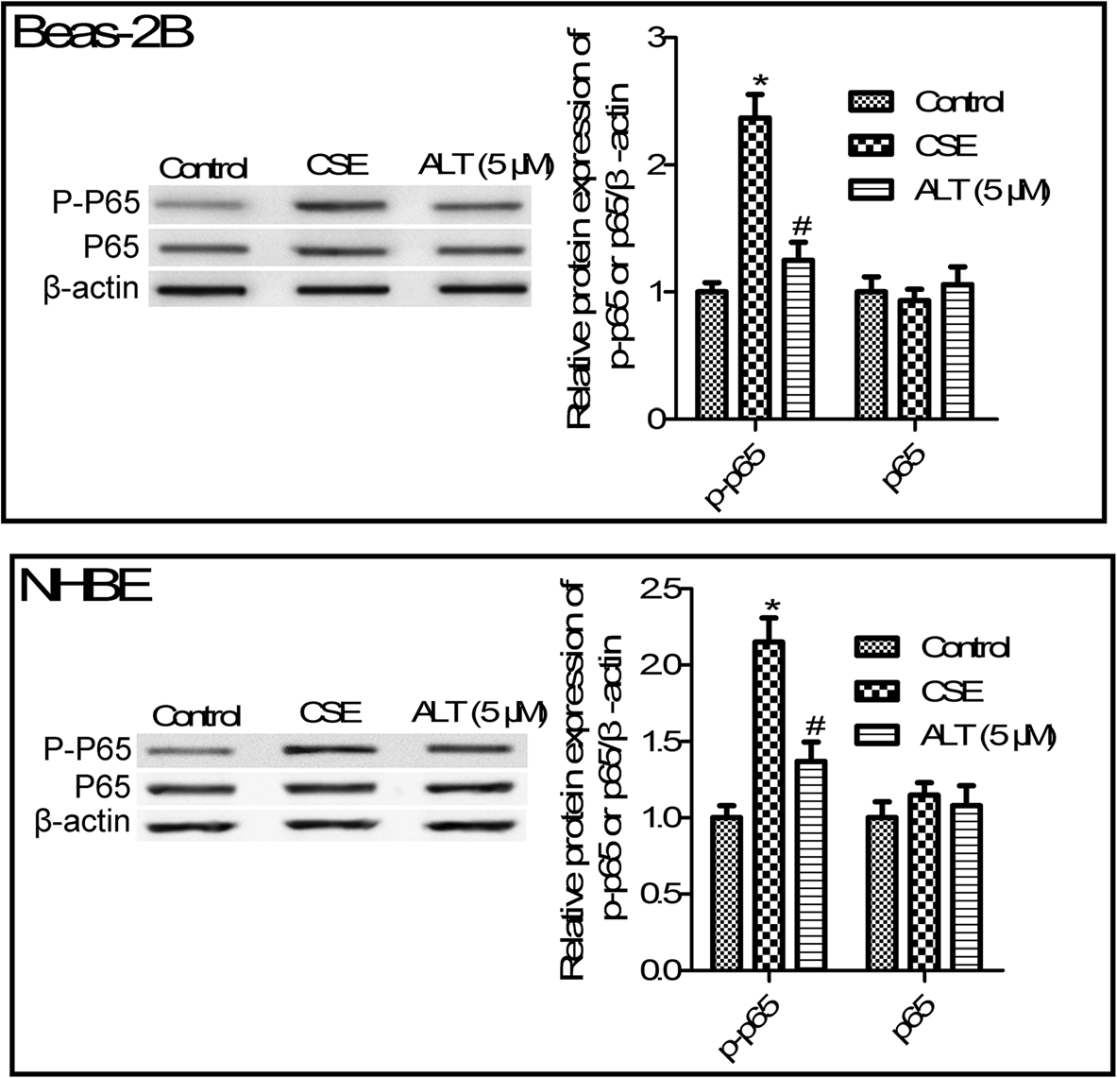
ALT inhibited CSE induced - NF-κB pathways activation in Beas-2B and NHBE cells. Western blot was used to determine protein expression of p-p65 and total p65. Beas-2B and NHBE cells were pre-treated with 5 μM ALT for 2 h and then administrated with 5% CSE for 24 h. Data were expressed as mean ± S.D. from three independent experiments. ^*^P values < 0.05 compared with the control group, ^#^*P* values < 0.05 compared with the CSE group. CSE: cigarette smoke extract; ALT: Alantolactone

Abundant evidence has been gathered showing the involvement of NF-κB pathway in COPD and inflammation [26]. As shown in Fig. 6, we found that 5% CSE treatment significantly increased p-p65 protein expression compared with the control group (*p* < 0.05). Further studies suggested that 5 μM ALT administration greatly impaired the protein expression of p-p65 (Fig. 6, *p* < 0.05). However, total protein of p65 in Beas-2B and NHBE cells after CSE treatment or ALT administration have changed little. These results suggested that ALT treatment suppressed CSE-induced NF-κB pathways.

### ALT treatment increased Nrf2/HO-1 pathway activation in CSE induced Beas-2B and NHBE cells

**Fig. 7:**
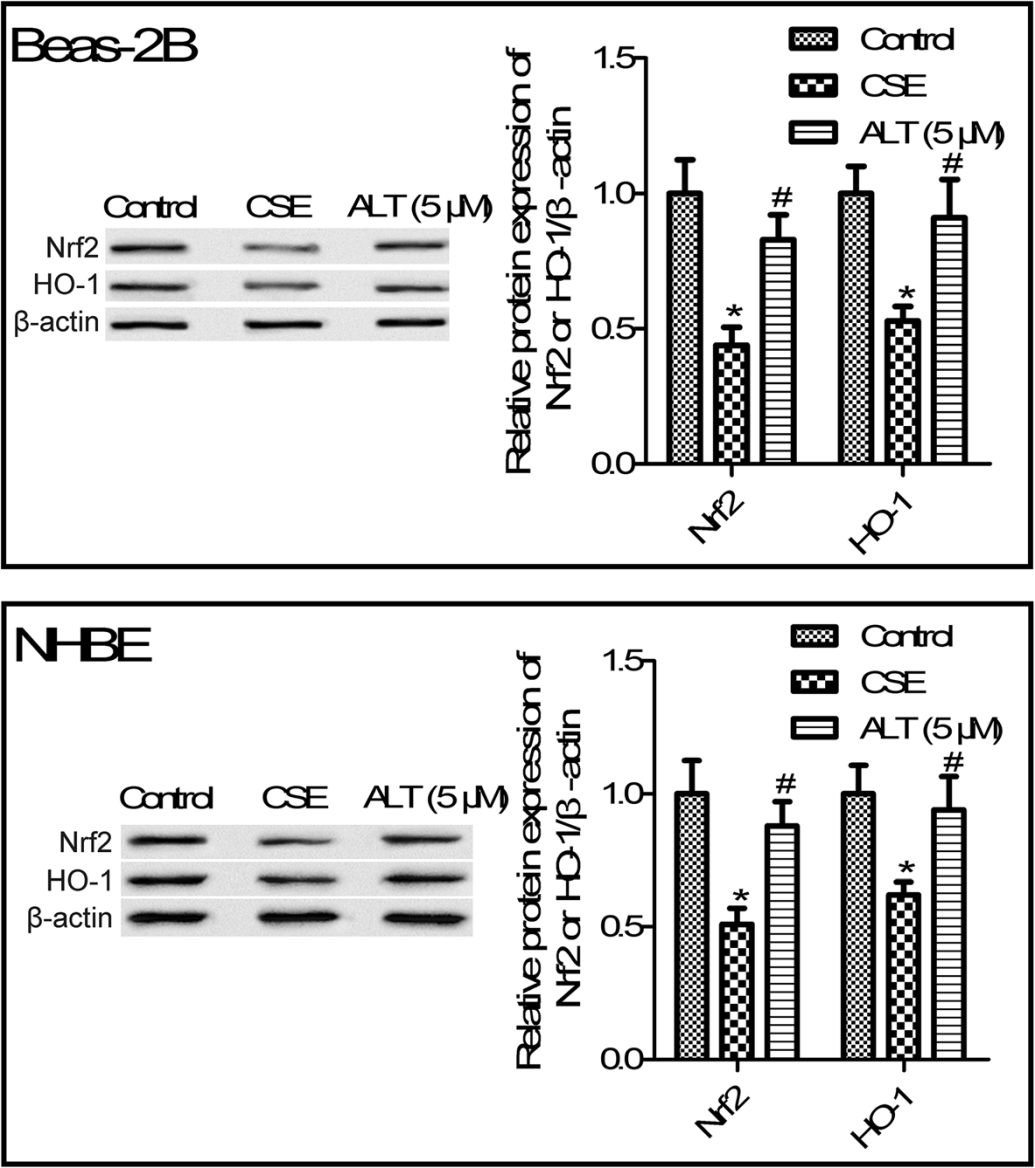
ALT promoted Nrf2/HO-1 activation in CSE induced Beas-2B and NHBE cells. Western blot was used to determine protein expression of Nrf2 and HO-1. Beas-2B and NHBE cells were pre-treated with 5 μM ALT for 2 h and then administrated with 5% CSE for 24 h. Data were expressed as mean ± S.D. from three independent experiments. ^*^P values < 0.05 compared with the control group, ^#^*P* values < 0.05 compared with the CSE group. CSE: cigarette smoke extract; ALT: Alantolactone

Nrf2/HO-1 pathway has been shown to play an important role in COPD [18]. Further studies suggested that 5% CSE exposure suppressed the protein expression of Nrf2 and the downstream HO-1 gene (Fig. 7, *p* < 0.05). We also observed that 1, 5 and 10 μM ALT treatment obviously increased Nrf2 and HO-1 protein expression compared with the CSE group. Moreover, 5 and 10 μM ALT exposure leads to a statistical significance between the CSE group and ALT treatment (Fig. 7, *p* < 0.05).

### HO-1 inhibitor reversed the effects of ALT on CSE-induced human bronchial epithelial cells

**Fig. 8:**
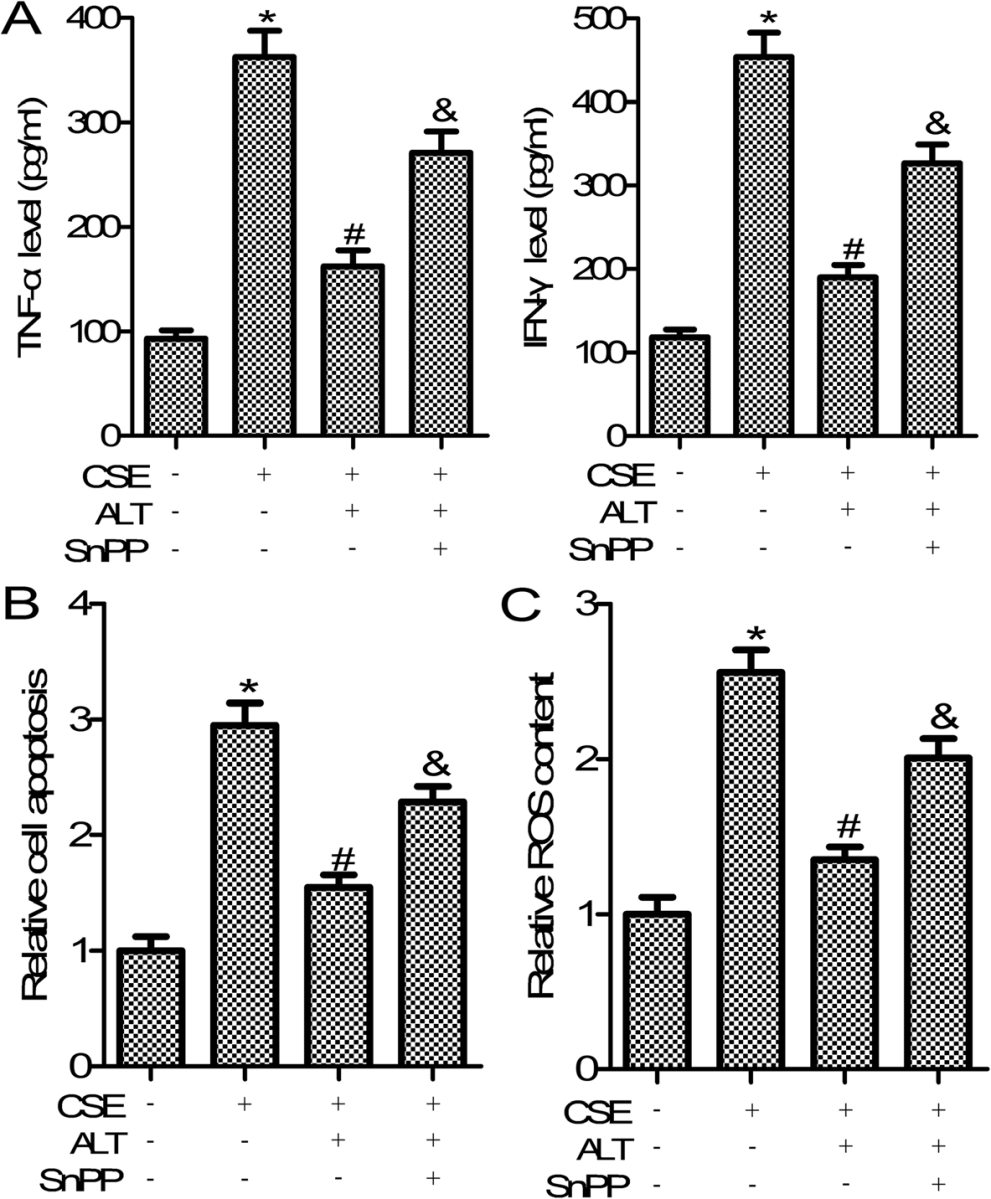
HO-1 inhibitor SnPP reversed the effects of ALT on CSE induced Beas-2B cells. **a** The commercially available ELISA kits were used to measure the level of TNF-α and IFN-γ; **b** Cell Death Detection PLUS ELISA assay was used to detect cell apoptosis; **c** DCFH-DA method was used to measure ROS production. Beas-2B cells were pre-treated with 5 μM ALT and SnPP (20 μM) for 2 h and then administrated with 5% CSE for 24 h. Data were expressed as mean ± S.D. from three independent experiments. ^*^P values < 0.05 compared with the control group, ^#^*P* values < 0.05 compared with the CSE group. CSE: cigarette smoke extract; ALT: Alantolactone

To analyse the function of the Nrf2/HO-1 signal pathway on the action of ALT in CSE-induced human bronchial epithelial cells, the HO-1 inhibitor SnPP was used. As shown in Fig. 8 a, SnPP treatment abrogated the inhibitory effects of ALT on TNF-α and IFN-γ secretion in CSE-induced Beas-2B cells. Moreover, we confirmed that SnPP treatment partial reversed the effects of ALT on CSE induced Beas-2B cell apoptosis and ROS production (Fig. 8 b and c). We also confirmed that SNPP also plays a similar role in another human bronchial epithelial cell line NHBE cells’ inflammatory response, apoptosis and oxidative stress (data not shown). These results suggested that ALT plays roles in CSE-induced Beas-2B and NHBE cell inflammation, apoptosis and oxidative stress, possibly through the Nrf2/HO-1 and NF-κB pathways.

**Fig. 9:**
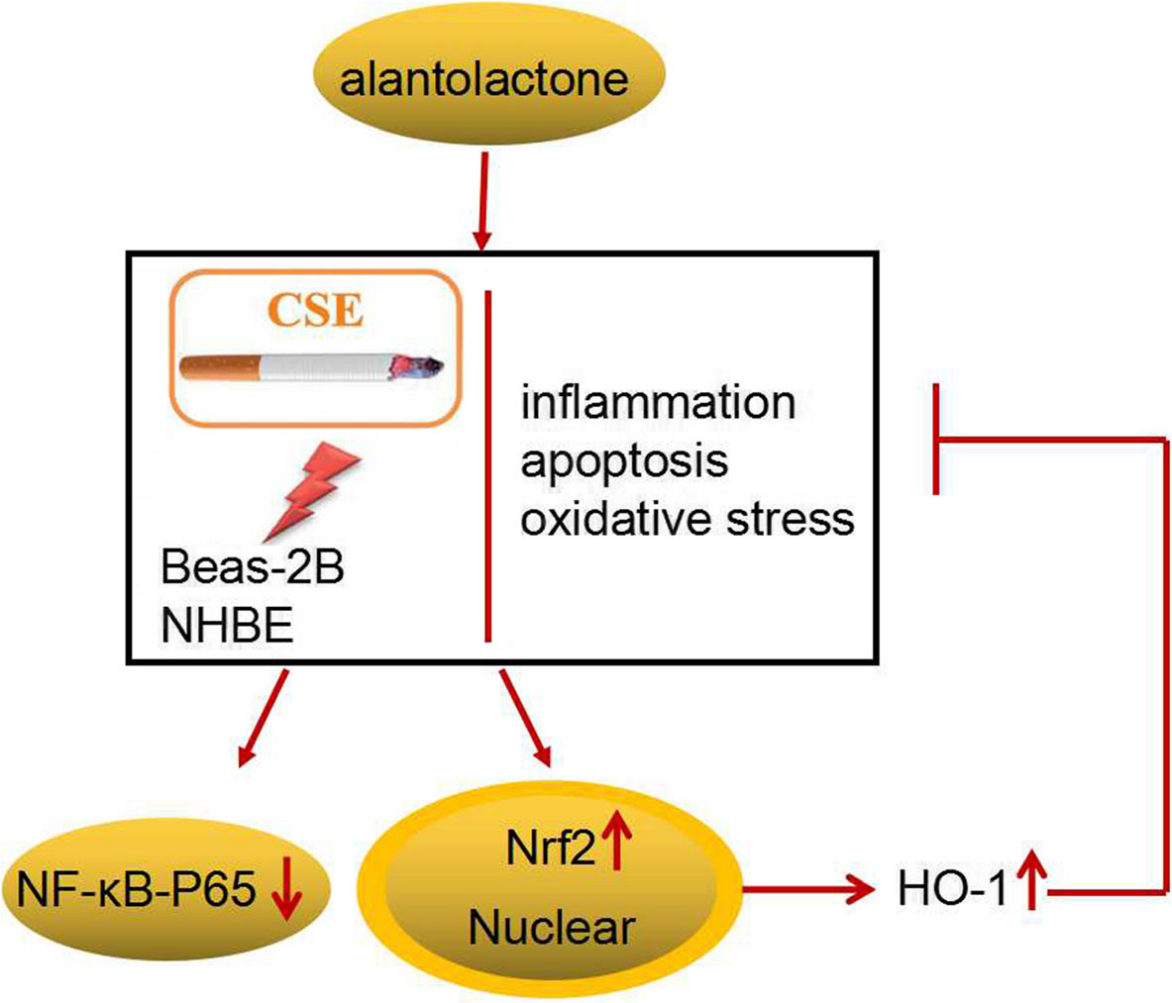
A schematic diagram of function and mechnism of ALT in CSE-induced human bronchial epithelial cells

## Discussion

COPD is considered the fourth leading cause of mortality globally [27]. Evidence has indicated that surgical therapy, rehabilitation and some agents, including methylxanthines, anticholinergics, corticosteroids, β2-agonists as well as phosphodiesterase-4 inhibitors contribute to reducing COPD symptoms and improving patients’ life quality [28]. However, no curative therapy for COPD has been achieved. CSE exposure induced persistent inflammation, oxidative stress and abnormal cell repair and apoptosis [29, 30]. In the present study, to further explore therapeutic targets and related drugs for COPD, we used CSE-treated human bronchial epithelial cells to mimic the COPD microenvironment in vitro.

Airway injury and repair are often associated with COPD, and apoptosis of airway epithelial cells is involved in this process. Evidence has suggested that airway epithelial cell apoptosis play roles in COPD [31, 32]. In the present study, we observed that alantolactone (ALT) treatment significantly suppressed CSE-induced Beas-2B and NHBE cell apoptosis. The sesquiterpene lactone alantolactone (ALT) has been isolated from *Inula helenium* and may exert anti-inflammatory properties in disease [33]. Muhammad Khan et al. observed that ALT exerted anti-oxidant effects through inhibiting reactive oxygen species (ROS) production [11]. However, the specific role and associated molecular mechanism is still unknown. In the current study, we observed that CSE in human bronchial epithelial cell lines Beas-2B and NHBE induced inflammation, apoptosis and oxidative stress. ALT treatment markedly impeded CSE-mediated inflammatory response, apoptosis and oxidative stress. ALT treatment markedly altered inflammatory cytokines IL-1 β, TNF-α, IL-6 and IFN-γ expression, cell apoptosis, caspase-3 activity, and levels of oxidative stress markers MDA, ROS and SOD.

Abundant evidence has been gathered showing ALT plays important roles in the NF-κB/COX-2-mediated signaling cascades and IKKβ kinase activity [34]. The nuclear factor-κB (NF-κB) pathway has been shown to play critical roles in COPD pathogenesis [35]. Sun et al. suggested that CSE treatment activates the NF-κB pathway in COPD mice and in RAW264.7 macrophages [36]. In agreement with the earlier reports, the present study confirmed that ALT treatment markedly suppressed NF-κB p-P65 protein expression in human bronchial epithelial cell line Beas-2B and NHBE cells. Moreover, ample evidence confirmed that Nrf2/ HO-1 signal pathway was implicated in oxidative stress response [37]. Nrf2/HO-1 has been shown to play a pivotal role in the inhibition of inflammation in COPD [18]. The regulation of CSE on Nrf2/HO-1 pathway is controversial [38, 39]. Some evidence suggested that CSE can promote the expression of Nrf2 and HO-1. On the other hand, it has also been proved that CSE can inhibit the expression of Nrf2 and HO-1 [18, 40]. The prsent study confirmed that CSE treatment inhibited the expression of NRf2. Interestingly, Ji Yeon Seo et al. found that ALT stimulated the nuclear accumulation of Nrf2 expression [41]. Consistent with these previous reports, we found that ALT treatment markedly increased Nrf2 and its downstream gene HO-1 expression in Beas-2B and NHBE cells. Finally, HO-1 inhibitor SnPP treatment partially reversed the effects of ALT on human bronchial epithelial cell inflammation, apoptosis and oxidative stress, as shown in Fig. 9.

## Conclusion

In conclusion, the current study showed that ALT treatment impeded CSE-induced Beas-2B and NHBE cell inflammation, apoptosis and oxidative stress. Also, we observed that ALT exposure suppressed NF-κB activation and increased Nrf2/HO-1 pathway activation. This study confirmed an effective role of ALT in CSE- exposed Beas-2B and NHBE cell in vitro; however, the specific role and potential molecular mechanism of ALT in COPD also needs to be explored in patient and animal studies.

## Data Availability

Not applicable.

## Abbreviations

ALT: Alantolactone
COPD: Chronic obstructive pulmonary disease
CS: Cigarette smoking
Nrf2: Nuclear factor erythroid-2-related factor-2
HO-1: Heme oxygenase-1
CSE: Cigarette smoke extract
MDA: Malondialdehyde
SOD: Superoxide dismutase
ROS: Reactive oxygen species
